# Press Releases Issued by Supplements Industry Organisations and Non-Industry Organisations in Response to Publication of Clinical Research Findings: A Case-Control Study

**DOI:** 10.1371/journal.pone.0101533

**Published:** 2014-07-03

**Authors:** Michael T. M. Wang, Greg Gamble, Mark J. Bolland, Andrew Grey

**Affiliations:** Department of Medicine, University of Auckland, Auckland, New Zealand; Copenhagen University Hospital, Denmark

## Abstract

**Background:**

Dietary supplement use is increasing despite lack of evidence of benefits, or evidence of harm. Press releases issued by the supplements industry might contribute to this situation by using ‘spin’ (strategies to hype or denigrate findings) to distort the results of clinical studies. We assessed press releases issued in response to publication of clinical studies on dietary supplements.

**Methods and Findings:**

We analyzed 47 supplements industry press releases and 91 non-industry press releases and news stories, generated in response to 46 clinical studies of dietary supplements published between 1/1/2005 and 5/31/2013. The primary outcome was ‘spin’ content and direction. We also assessed disposition towards use of dietary supplements, reporting of study information, and dissemination of industry press releases. More supplements industry press releases (100%) contained ‘spin’ than non-industry media documents (55%, P<0.001). Hyping ‘spin’ scores were higher in industry than non-industry media documents for studies reporting benefit of supplements (median ‘spin’ score 3.3, 95% CI 1.0–5.5 vs 0.5, 0–1.0; P<0.001). Denigratory ‘spin’ scores were higher in industry than non-industry media documents for studies reporting no effect (6.0, 5.0–7.0 vs 0, 0–0; P<0.001) or harm (6.0, 5.5–7.5 vs 0, 0–0.5; P<0.001) from a supplement. Industry press releases advocated supplement use in response to >90% of studies that reported no benefit, or harm, of the supplement. Industry press releases less frequently reported study outcomes, sample size, and estimates of effect size than non-industry media documents (all P<0.001), particularly for studies that reported no benefit of supplements. Industry press releases were referenced by 148 news stories on the websites of 6 organizations that inform manufacturers, retailers and consumers of supplements.

**Conclusions:**

Dietary supplements industry press releases issued in response to clinical research findings are characterized by ‘spin’ that hypes results that are favourable to supplement use and denigrates results that are not.

## Introduction

About half of US adults, and two-thirds of those>60 years, take dietary supplements [1]. Similar data have been reported outside of the USA [2], [3]. The supplements industry is profitable: Americans spend more than US$30 billion annually on dietary supplements [1]. Motivations to take dietary supplements are diverse, but users most commonly cite a wish to improve or maintain health [1], [4]. The sources of information which influence decisions to use dietary supplements are also multiple. Only 23% of US adults who take supplements do so on the advice of a health care professional [1]. In healthy older adults, important sources of information that influence decisions about supplement use include magazines, news articles, and people other than health professionals [2], [5].

In the past decade, there has been intensive investigation of the health benefits and risks of dietary supplements. Consequently, there have been many publications in prominent medical journals on dietary supplements; these often reported no benefit and sometimes reported harm [1], [6]–[8]. Publications of randomized clinical trial data showing no health benefit of omega-3 fatty acids had no discernible effect on the contemporaneous progressive increase in use of the supplements [9]. The reason(s) for the burgeoning use of dietary supplements despite accrual of rigorous evidence of no benefit or harm is uncertain, but a survey of supplement users reported that only 25% of users would alter their behaviour in response to findings of clinical studies that contradicted the health claims made by supplements manufacturers [4].

Organizations that represent the commercial interests of supplements manufacturers take an interest in the outcomes of clinical research on dietary supplements. A means by which organizations with commercial interests might influence the responses of supplements users to the outcomes of clinical research is via press releases that generate news stories in media accessed by marketers and consumers of supplements. To investigate this possibility, we analyzed the tone, content, conclusions, and propagation of press releases generated by prominent organizations representing the dietary supplements industry in response to the publication of clinical research about supplements. We also compared the industry press releases to contemporaneous non-industry press releases or news stories.

## Materials and Methods

### Study Documents

Between 5/31/13 and 6/15/13, we extracted from 3 industry websites (the Council for Responsible Nutrition, CRN, “the leading trade association representing dietary supplement manufacturers and ingredient suppliers”, http://www.crnusa.org, the Alliance for Natural Health,ANH, “a non-governmental organisation promoting natural and sustainable approaches to healthcare worldwide”, http://www.anh-usa.org, and the Natural Products Association,NPA, “the leading representative of the dietary supplement industry”, http://www.npainfo.org) press releases issued in response to clinical studies of dietary supplements published between 1/1/05 and 5/31/13. We defined a clinical study as one that assessed the effects on human health of a dietary supplement or its food-based equivalent.

For each publication that generated a press release from an industry source, we collated two press releases or news stories from non-industry sources, using a structured approach. First, we extracted press releases from the National Institutes Health (NIH)/National Center for Complementary and Alternative Medicine (NCCAM), by searching its website and the EurekAlert! database (http://www.eurekalert.org). Next, we collated press releases issued by the journals that published the source articles, by searching the journal websites and the EurekAlert! database. Lastly, we accessed news stories from news agencies, identified by a Google search using search terms that included the name of the supplement, the primary author of the source article, and the journal of publication, with a time limit of two months from the date of publication. We extracted the first 1–2 news stories that were identified by our search. If a press release referred to more than one source publication, it was analysed for each publication. If fewer than two corresponding non-industry media documents were identified, the industry press release was excluded from further analysis.

The publications that stimulated the press releases from industry sources were collated using PubMed.

**Figure 1:**
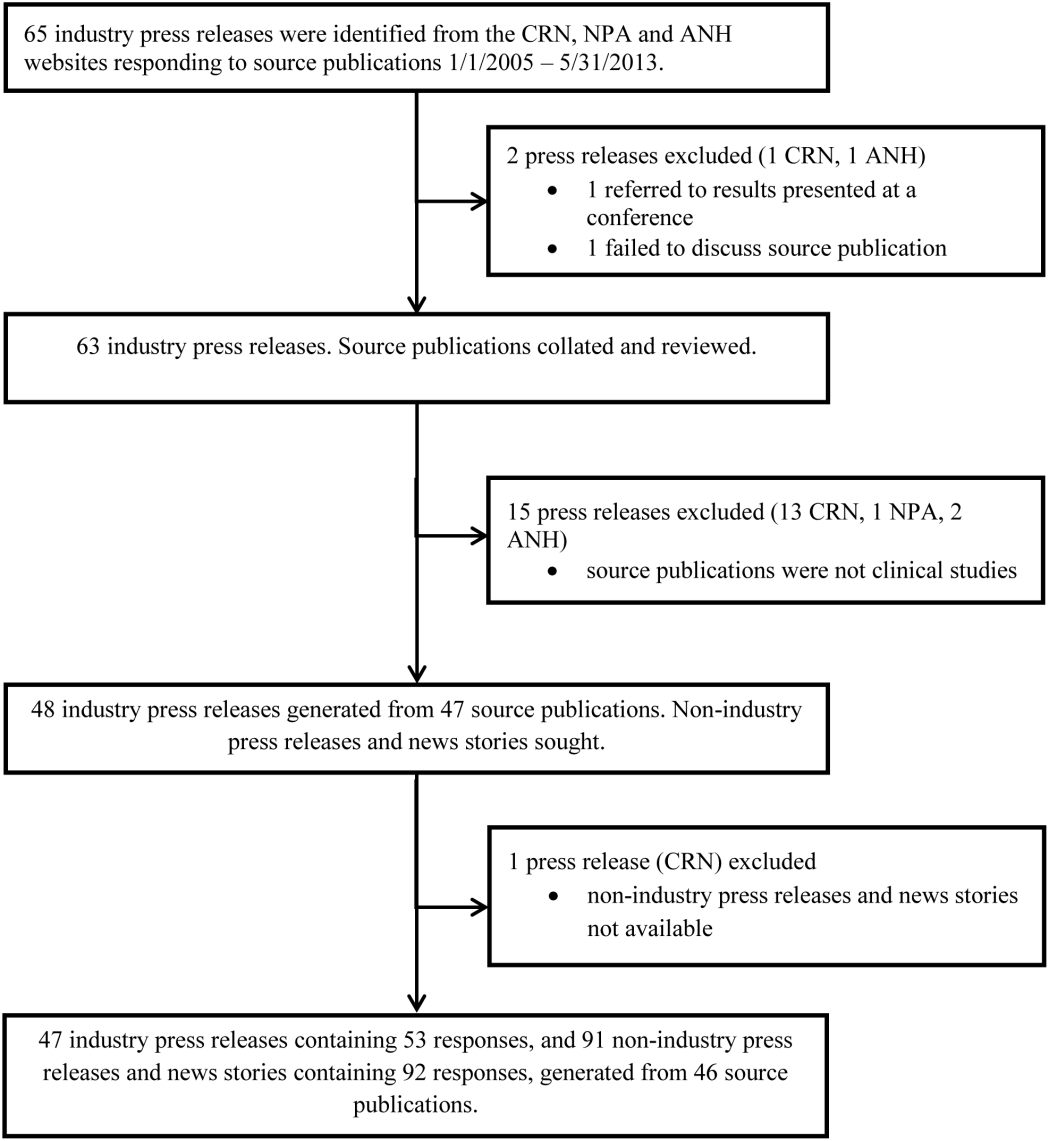
Collation of study documents. CRN, Council for Responsible Nutrition; NPA, Natural Products Association; ANH, Alliance for Natural Health.

Figure 1 summarizes the collation of study documents.

### Data Abstraction

Data were abstracted independently by two reviewers (MW and AG). For data that involved some subjectivity, such as the presence, type, and direction of ‘spin’, the interpretation of the title and text of the media documents and the ‘spin’ score, differences were resolved by consensus.

#### Source Publications

We adapted a data abstraction tool that evaluated the quality of reporting of clinical research [10], [11] (Text S1). Study conclusions were categorized as favours supplement use; does not favour supplement use - no effect; or does not favour supplement use - harm. If the source publication reported more than one outcome and the effects of the intervention were mixed, the assignation of study conclusion was to the category of the predominant effect or, if either benefit or harm was reported in combination with no effect, to favours supplement use or does not favour supplement use - harm, respectively.

#### Press Releases

From the press releases and news stories, data were extracted on reporting of study characteristics (Text S2). Quotes from industry staff, independent experts, and study investigators were collated. The title and text of each media document were assessed as to whether they supported use of the supplement, did not support use of the supplement, or were neutral [10]. If one reviewer considered a document to be neutral towards supplement use and the other reviewer considered it to be either supportive or not supportive of supplement use, the document was scored as supporting or not supporting use, respectively.

#### ‘Spin’ assessment

Each press release or news story was assessed for the presence and amount of ‘spin’ considered to be either hyping of, or denigratory towards, the source publication, using a standardized format (Text S3). Hyping ‘spin’ is specific reporting that unduly emphasizes or exaggerates the benefit of an experimental treatment [12]. We defined denigratory ‘spin’ as specific reporting that unduly downplays or dismisses the lack of benefit, or harm, caused by an experimental treatment. To assess hyping ‘spin’ we adapted published methods which included a checklist of ‘spin’ strategies [10], [13]. To assess denigratory ‘spin’ we designed a data abstraction form by adapting that used for assessment of hyping ‘spin’ and incorporating into the checklist commonly employed denigratory strategies identified from a review of relevant literature [14], [15] and media and academic responses to publication of clinical research.

Each press release and news story in the study sample set was independently assessed by two reviewers (MW and AG). If a document was considered to either hype or denigrate the clinical research, the assessor proceeded to complete a checklist of ‘spin’ techniques (Text S3). To produce a score for each form of ‘spin’, we assigned one point to each checklist item identified in a press release or news story. Summing the hyping and denigratory ‘spin’ scores produced a total ‘spin’ score. Discrepancies in the total ‘spin’ score of >2 points between reviewers were resolved by consensus. If the difference between the reviewers’ scores was≤2 points, the mean value of the scores was included in the analysis. The inter-observer agreement for the ‘spin’ score was assessed in a sample of 10 press releases and news stories (5 industry and 5 non-industry) that were independent from the final study sample, being issued prior to 1 January 2005. In this analysis, the kappa coefficient was 0.64 (95% CI 0.59–0.84). The kappa coefficient is the proportion of agreement between two assessors, adjusted for chance agreement [16]. Values within the range 0.61–0.80 indicate substantial agreement [17].

### Industry press release propagation

To assess the potential impact of the industry press releases, we sought news reports that referenced them. We searched the websites of six organizations (NewHope360, http://newhope360.com/; Nutritional Outlook, http://www.nutritionaloutlook.com/; Natural Products Insider, http://www.naturalproductsinsider.com/; Drug Store News, http://drugstorenews.com/; Nutraceuticals World, http://www.nutraceuticalsworld.com; Whole Food Magazine, http://www.wholefoodsmagazine.com/) that provide information and advice to manufacturers, marketers and retailers of dietary supplements, using search terms that included the sources of the industry press releases and each of the interventions studied in the source publications. From each site, we collated all news stories that directly referenced, or contained verbatim material from, any of the industry press releases in our sample set.

### Statistics

The sample size of industry press releases was pragmatically determined by the number issued during the study period. Analyses were performed using Graph Pad Prism version 6.02 (http://www.graphpad.com). Between-group comparisons of continuous variables were made using Wilcoxon rank sum text. Analyses of categorical data were performed using Fisher’s exact test test or chi-squared test, as appropriate. Confidence intervals about medians were calculated using Graph Pad Prism version 6.02 and about percentages using Open Source Epidemiologic Statistics for Public Health (http://www.openepi.com), accessed November 2013. Since all comparisons were pre-planned no adjustment for multiplicity was performed. All tests were two tailed and P<0.05 was considered significant. Data are presented as median (95% CI) unless otherwise stated.

## Results

### Industry press releases and source publications

The final dataset contained 47 industry press releases (39 from the Council for Responsible Nutrition (CRN),; 3 from the Natural Products Association (NPA); 5 from the Alliance for Natural Health (ANH)). Some industry press releases referred to more than one source article, and some source articles generated press releases from more than one industry source. Consequently, the 47 industry press releases contained 53 responses to 46 source publications [18]–[63] (Table 1). Thirty-eight (83%) of the source articles were published in the seven most prestigious internal medicine journals, as judged by impact factor. The median (range) impact factor of the journals in which the source articles were published was 11 (4–51). Thirty-nine (85%) studies reported “hard” disease outcomes.

**Table 1:** Press releases, news stories and source publications.

Sources of Press Releases and News Stories		Source Article	Study Design	N	Intervention/Exposure	Reported Outcome Measure(s)	Results
Industry	Non-industry						
CRN	NHLBI, JAMA	Lee 2005 [18]	RCT	39876	Vitamin E	First majorCV event	No effect
						Total invasivecancer	No effect
CRN	JAMA, CBC	Lonn 2005 [19]	RCT	9541	Vitamin E	Cancer incidence	No effect
						Cancer mortality	No effect
						Major CV events	No effect
CRN	BBC, NY Times	Miller 2005 [20]	Meta-RCT	135967	Vitamin E	All-cause mortality	Harm
CRN	NY Times, SF Chronicle	Bent 2006 [21]	RCT	225	Saw palmetto	Urologicalsymptoms	No effect
						Urinary flowrate	No effect
CRN	Health Day, NY Times	Bonaa 2006 [22]	RCT	3749	Folic acid + Vitamin B_12_+ Vitamin B_6_	CV disease	No effect
					Folic acid + Vitamin B_12_		No effect
					Vitamin B_6_		No effect
CRN	NCCAM, NY Times	Clegg 2006 [23]	RCT	1583	Chondroitin	Knee pain	No effect
					Glucosamine		No effect
					Chondroitin + glucosamine		No effect
CRN	NHLBI, NY Times	Jackson 2006 [24]	RCT	36282	Calcium + Vitamin D	Hip fracture	No effect
						Spine fracture	No effect
						Total fracture	No effect
CRN	NY Times, Washington Post	Lonn 2006 [25]	RCT	5522	Folic acid + Vitamin B_6_+ Vitamin B_12_	Mortality fromCV causes,myocardialinfarction orstroke	No effect
CRN	JAMA, Wall Street Journal	Prince 2006 [26]	RCT	1460	Calcium	Osteoporoticfractures	No effect
						Vertebraldeformity	No effect
CRN	NHLBI, NY Times	Wactawski-Wende 2006 [27]	RCT	36282	Calcium + Vitamin D	Colorectalcancer^i^	No effect
CRN	JAMA, AP	Bjelakovic 2007 [28]	Meta-RCT	232606	Beta carotene	All-cause mortality	Harm
					Vitamin A		Harm
					Vitamin C		No effect
					Vitamin E		Harm
					Selenium		No effect
CRN	JAMA, AP	Cole 2007 [29]	RCT	1021	Folic acid	Colorectaladenoma	No effect
CRN	National Post, US News	Lappe 2007 [30]	RCT	1179	Calcium	Cancer^i^	No effect
					Calcium + Vitamin D		Benefit
CRN	JAMA, NY Times	Lin 2007 [31]	Prospective Cohort	31487	Calcium + Vitamin D	Breast Cancer	Benefit
CRN	NY Times, US News	Reichenbach 2007 [32]	Meta-RCT	3846	Chondroitin	Joint pain	No effect
CRN	BBC, NY Times	Shah 2007 [33]	Meta-RCT	1356	Echinacea	Incidence ofcommon cold	Benefit
				1630		Duration ofcommon cold	Benefit
CRN, ANH	Cochrane, ABC	Bjelakovic 2008 [34]	Meta-RCT	232550	Beta-carotene	Mortality	Harm
					Vitamin A		Harm
					Vitamin C		No effect
					Vitamin E		Harm
					Selenium		No effect
CRN	NCCAM, USA Today	Sawitzke 2008 [35]	RCT	572	Glucosamine	Joint spacewidth	No effect
					Chondroitin		No effect
					Glucosamine + Chondroitin		No effect
CRN	JAMA, Reuters	Sesso 2008 [36]	RCT	14641	Vitamin E	Major CVevents	No effect
					Vitamin C		No effect
CRN, ANH	JAMA, Bloomberg	Christen 2009 [37]	RCT	5442	Folic acid + Vitamin B_6_+Vitamin B_12_	Total age-relatedmaculardegeneration^ii^	Benefit
						Visually significantage-related maculardegeneration^i^	Benefit
CRN	JAMA, Reuters	Ebbing 2009 [38]	RCT	6837	Folic acid + Vitamin B_12_	Cancer incidence	Harm
						Cancer mortality	Harm
						All-cause mortality	Harm
					Vitamin B_6_	Cancer incidence	No effect
						Cancer mortality	No effect
						All-cause mortality	No effect
CRN, NPA	CNN, Health Day	Ginde 2009 [39]	Cross-sectional cohort	18883	Vitamin D	Upper respiratory tract infection	Benefit
ANH	JNCI, Reuters	Lin 2009 [40]	RCT	7627	Vitamin C	Invasive cancer^ii^	No effect
						Cancer mortality^ii^	No effect
					Vitamin E	Invasive cancer^ii^	No effect
						Cancer mortality^ii^	No effect
					Beta Carotene	Invasive cancer^ii^	No effect
						Cancer mortality^ii^	No effect
CRN, ANH	JAMA, CNN	Neuhouser 2009 [41]	Prospective Cohort	161808	Multivitamins	Cancer	No effect
						CV disease	No effect
						Total mortality	No effect
CRN, ANH	JAMA, NY Times	Park 2009 [42]	Prospective Cohort	492810	Dairy food intake	Cancers of thedigestive system	Benefit
					Calcium intake	Total cancer	Benefit
						Cancers of thedigestive system	Benefit
CRN	JAMA, Reuters	Snitz 2009 [43]	RCT	3069	Gingko biloba	Cognitivefunction	No effect
CRN	JAMA, Reuters	Armitage 2010 [44]	RCT	12064	Folic acid + Vitamin B_12_	First majorvascular event	No effect
CRN	BMJ, Reuters	Bolland 2010 [45]	Meta-RCT	11921	Calcium supplements	Myocardialinfarction	Harm
						Stroke	No effect
						Myocardialinfarction, stroke,or sudden death	No effect
CRN	JAMA, NY Times	Makrides 2010 [46]	RCT	Women 2363	DHA	Maternaldepression	No effect
				Children 726		Infant cognitionand languagedevelopment	No effect
CRN	JAMA, ABC	Quinn 2010 [47]	RCT	402	DHA	Cognitivefunction	No effect
CRN	ABC, Reuters	Wandel 2010 [48]	Meta-RCT	3803	Chondroitin	Joint pain	No effect
					Glucosamine		No effect
					Chondroitin + glucosamine		No effect
CRN	NCCAM, JAMA	Barry 2011 [49]	RCT	369	Saw palmetto	Lower urinarytract symptoms	No effect
CRN	NCCAM, JAMA	Klein 2011 [50]	RCT	35533	Selenium	Prostate cancer	No effect
					Vitamin E		Harm
					Selenium + Vitamin E		No effect
CRN, ANH	JAMA, Reuters	Mursu 2011 [51]	Prospective cohort	38772	Multivitamins	Mortality	Harm
					Vitamin B_6_		Harm
					Folic acid		Harm
					Iron		Harm
					Magnesium		Harm
					Zinc		Harm
					Copper		Harm
					Calcium		Benefit
CRN	Health Day, Reuters	Bischoff-Ferrari 2012 [52]	Meta-RCT	31022	Vitamin D	Hip fractures	Benefit^a^
						Non-vertebralfractures	Benefit^a^
CRN	JAMA, Reuters	Gaziano 2012 [53]	RCT	14641	Multivitamin	Total cancer	Benefit
CRN	Health Day, NPR	Kramer 2012 [54]	Prospective Cohort	15099	Vitamin D	All-cause mortality	No effect
CRN, NPA	ABC, LA Times	Kwak 2012 [55]	Meta-RCT	20485	Omega-3 fatty acids	CV events	No effect
CRN, NPA	Guardian (UK), Health Day	Li 2012 [56]	Prospective Cohort	23980	Dietary andsupplemental calcium	Myocardialinfarction	Harm
						Stroke	No effect
						CV mortality	No effect
CRN	JAMA, Reuters	Murdoch 2012 [57]	RCT	322	Vitamin D	Upper respiratorytract infection	No effect
CRN	JAMA, Reuters	Rizos 2012 [58]	Meta-RCT	68680	Omega-3 fatty acid	All-causemortality	No effect
						Cardiac mortality	No effect
						Sudden death	No effect
						Myocardialinfarction	No effect
						Stroke	No effect
CRN	JAMA, Reuters	Sesso 2012 [59]	RCT	14641	Multivitamin	Major CVevents	No effect
CRN	NEI, Reuters	The AREDS2 Research Group 2013 [60]	RCT	4203	Lutein + zeaxanthin	Cataract Surgeryiii	No effect
						Vision Lossiii	No effect
CRN	NEI, JAMA	The AREDS2 Research Group 2013 [61]	RCT	4203	Lutein + zeaxanthin	Maculardegeneration	No effect
					DHA +EPA		No effect
					Lutein + zeaxanthin +DHA + EPA		No effect
CRN	BMJ, Daily Express	Michaelsson 2013 [62]	Prospective Cohort	61433	Dietary andsupplemental calcium	All-causemortality	Harm
						Cause specificCV disease mortality	Harm
						Ischaemic heartdisease mortality	Harm
						Stroke mortality	No effect
CRN, NPA	JAMA, Reuters	Xiao 2013 [63]	Prospective Cohort	388229	Dietary andsupplemental calcium	CV diseasemortality	Harm^iv^
						Heart diseasemortality	Harm^iv^
						Cerebrovasculardisease mortality	No effect

RCT, Randomized Controlled Trial; Meta-RCT, Meta-analysis of Randomized Controlled Trials; CV, Cardiovascular; DHA, docosahexanoic acid; EPA, eicosapentanoic acid.

CRN, Council for Responsible Nutrition; ANH, Alliance for Natural Health; NPA, Natural Products Association; NCCAM, National Centre for Complementary and Alternative Medicine; NHLBI, National Heart, Lung and Blood Institute; NCI, National Cancer Institute; NEI, National Eye Institute; JAMA, JAMA Network; BMJ, British Medical Journal; Cochrane, Cochrane Collaboration; JNCI, Journal of the National Cancer Institute; Reuters, Reuters Health; NY Times, New York Times; ABC, American Broadcasting Company; AP, Associated Press; BBC, British Broadcasting Corporation; CNN, Cable News Network; US News, US News & World Report; CBC, Canadian Broadcasting Corporation; LA Times, Los Angeles Times; NPR, National Public Radio; SF Chronicle, San Francisco Chronicle.

i Source article reports on a secondary outcome. The primary outcome of the study is fractures.

ii Source article reports on secondary outcomes. The primary outcome of the study is cardiovascular disease.

iii Source article reports on secondary outcomes. The primary outcome of the study is advanced age-related macular degeneration.

iv Harm reported in men, no effect was observed for women.

a Treatment-adherence analysis. Intention to treat analyses found no treatment effect on hip fracture and significant reduction in non-vertebral fracture.

Table S1 summarizes the types of studies reported by the source publications, according to study outcomes. Most of the studies (37/46, 80%) were randomized trials or meta-analyses of randomized trials, and most (28/46, 61%) reported no effect of the supplement investigated.

### Non-industry press releases and news stories

Table 1 contains the sources of the non-industry press releases and news stories. One non-industry media document referred to two source articles. Consequently, there were 91 non-industry press releases and news stories, containing 92 responses to the 46 source publications. 55 (60%) of the non-industry press releases and news stories were from news agencies, 28 (31%) from the journal of publication, and 8 (9%) from the National Institutes of Health (NIH)/National Center for Complementary and Alternative Medicine (NCCAM).

### ‘Spin’ analyses

At least 1 item of ‘spin’ was present in all 53 industry media responses, compared to 51/92 (55%) non-industry media responses (P<0.001). The prevalence of hyping ‘spin’ did not differ between industry and non-industry media responses (16/53, 30%, vs 38/92, 41%; P = 0.21) but denigratory spin was more frequent in the industry media responses (40/53, 75% vs 21/92, 23%; P<0.001).

**Figure 2:**
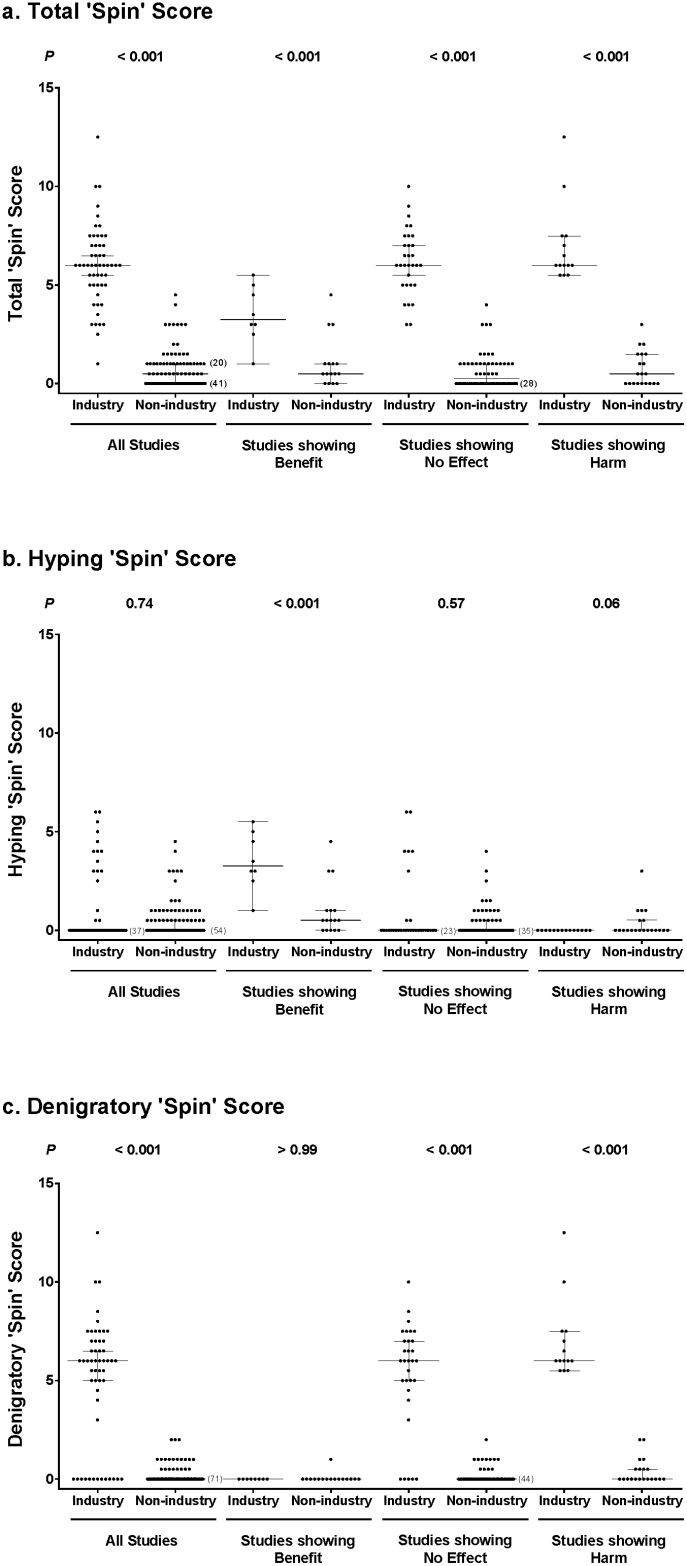
Total (a), hyping (b) and denigratory (c) ‘spin’ scores of industry and non-industry media responses, according to outcomes of source publications. Each point represents the ‘spin’ score of an individual media response. Where multiple scores of the same value occur, the number of overlapping ‘spin’ scores is indicated in parentheses to the right of the overlapping values. Bars represent the median and 95% CI.

‘Spin’ scores differed by >2 points between reviewers for 19/145 (13%) of media responses, 13/53 industry and 6/92 non-industry. Figure 2a shows the total ‘spin’ scores in the industry and non-industry media responses. Considering all studies, the ‘spin’ score in the industry media responses (median 6, 95%CI 5.5–6.5) was higher than that in the non-industry media responses (0.5, 0.0–1.0; P<0.001). ‘Spin’ scores were higher in the industry media responses for studies that reported benefit, reported no benefit, or reported harm of the supplement (P<0.001 for each).

To investigate whether directional ‘spin’ was present, we analyzed the hyping and denigratory ‘spin’ scores according to the outcome of the study (Figures 2b–c). Considering all studies, hyping ‘spin’ scores were similar between industry and non-industry media responses (P = 0.74). For studies that reported benefit of supplements, hyping ‘spin’ scores were higher in industry media responses (3.3, 1.0–5.5) than in the non-industry media responses (0.5, 0–1.0; P<0.001). Hyping ‘spin’ scores were not different between industry and non-industry media responses for studies that reported no effect (P = 0.57) or harm (P = 0.06). Considering all studies, the denigratory ‘spin’ score was higher in industry media responses (6, 5.0–6.5) than non-industry media responses (0, 0–0; P<0.001). No denigratory ‘spin’ was present in industry media responses to studies reporting benefit of a supplement. Denigratory ‘spin’ scores were significantly higher in industry than non-industry media responses to publication of studies that found no effect (6.0, 5.0–7.0 vs 0, 0–0; P<0.001) or harm (6.0, 5.5–7.5 vs 0, 0–0.5; P<0.001) from a supplement. Removing responses from any of the non-industry sources (journals of publication, NIH, or news agencies) did not change the results of any of these analyses (data not shown).

The frequency of ‘spin’ techniques that were identified in at least 15% of media responses from either industry or non-industry sources is shown in Table S2.

### Interpretation of press releases and news stories

**Figure 3:**
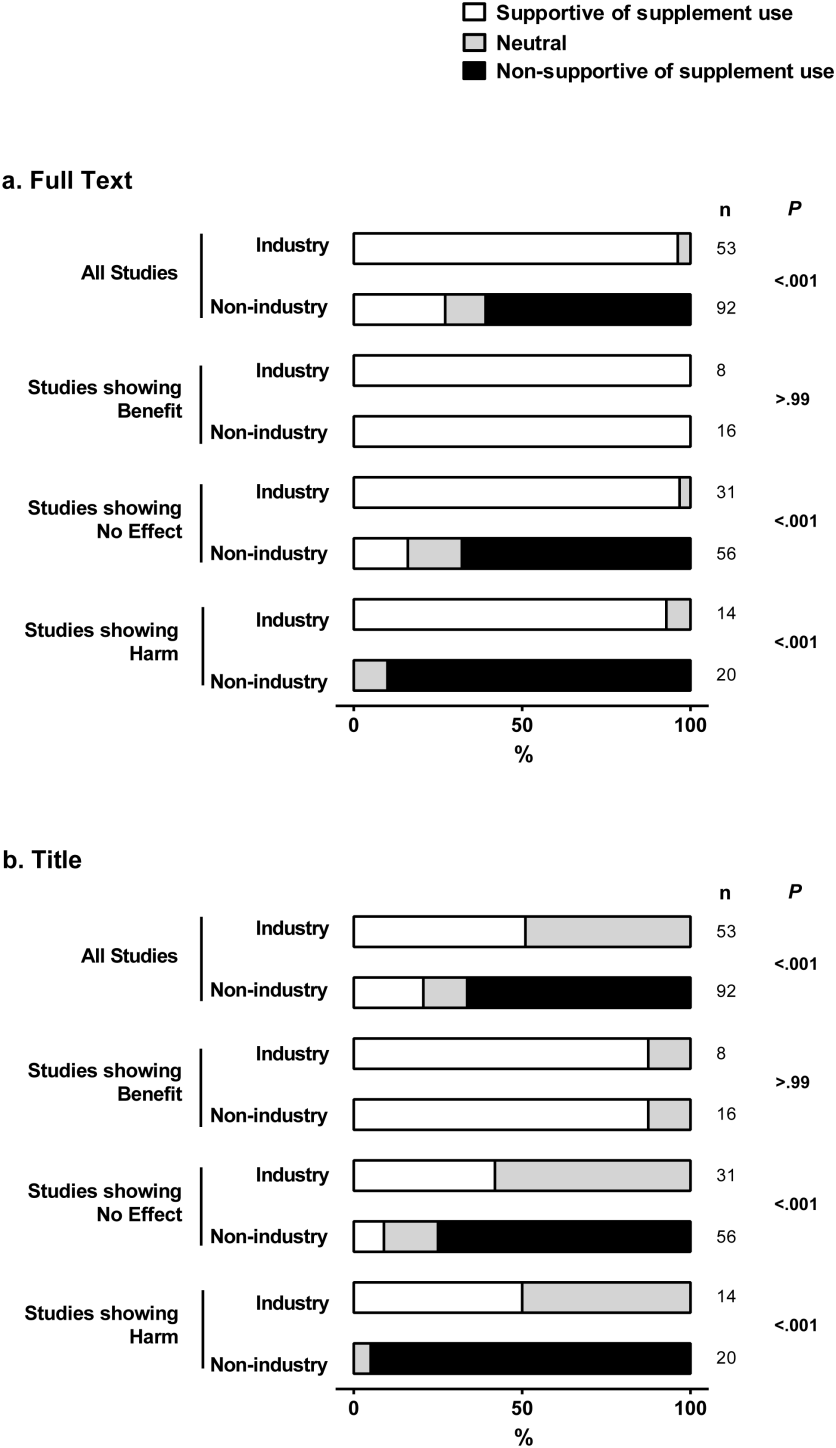
Disposition of industry and non-industry media responses towards use of supplements, according to outcome of source publications. Data on the bars are the percentage of full text (a) and titles (b) of media responses that are supportive, non-supportive or neutral towards supplement use.

There was no instance of assessor disagreement over the disposition of the media documents towards use of supplements. Industry media responses were more likely than non-industry media responses to support use of supplements. Considering all studies, the text of 51/53 (96%, 87–100) industry media responses was supportive of use of the supplement(s), in comparison to 25/92 (27%, 19–37) non-industry media responses (P<0.001) (Figure 3a). Industry and non-industry media responses were equally supportive of use of the supplement(s) in studies which reported benefit. For studies reporting no effect or harm of a supplement, the text of 97% (83–100) and 93% (66–100), respectively, of industry media responses was supportive of use of the supplement, while 16% (8–28) and 0% (0–17) of non-industry media responses were supportive of use (P<0.001 for each industry vs non-industry comparison). A similar pattern of results was apparent when the titles of media responses were assessed (Figure 3b).

### Reporting of study characteristics and outcomes

Reporting of study design did not differ between industry and non-industry media responses, but industry media responses were less likely than non-industry media responses to report sample size (18/53, 34% vs 88/93, 96%; P<0.001) or to identify study outcomes (33/53, 62% vs 92/92, 100%; P<0.001) (Table 2). Each of these differences was more marked for studies that reported no effect or harm of a supplement. Study outcomes were identified in 8/8 (100%) industry media responses to studies reporting benefit of supplements, but only 19/31 (61%) and 6/14 (43%) of those issued in response to studies reporting no effect or harm, respectively. Study outcomes were reported using relative numbers in 9/53 (17%) of industry media responses and 39/92 (42%) of non-industry media responses (P = 0.002); outcomes were reported using absolute numbers in 0/53 (0%) of industry media responses and 25/92 (27%) of non-industry media responses (P<0.001). A numerical description of the study outcome was present in 7/8 (88%) industry media responses to studies reporting benefit of a supplement, but only 2/31 (6%) and 1/14 (7%) media responses to studies reporting no effect or harm, respectively.

**Table 2:** Reporting of study characteristics and outcomes.

Reporting of Study Characteristics		All Studies			Studies Reporting Benefit		Studies Reporting No Effect		Studies Reporting Harm	
		Industry (n = 53)		Non-industry (n = 92)	Industry (n = 8)	Non-industry (N = 16)	Industry (n = 31)	Non-industry (n = 56)	Industry (n = 14)	Non-industry (n = 20)
Study Design	n		24	54	6	9	10	36	8	9
	%		45 (32–60)	59 (48–69)	75 (35–97)	56 (30–80)	32 (17–51)	64 (50–77)	57 (29–82)	45 (23–68)
	P		0.12		0.66		0.007		0.73	
Sample Size	n		18	88	6	15	12	54	0	19
	%		34 (22–48)	96 (89–99)	75 (35–97)	94 (70–100)	39 (22–58)	96 (88–100)	0 (0–23)	95 (75–100)
	P		<0.001		0.25		<0.001		<0.001	
Study Outcome*Reported in Words*	n		33	92	8	16	19	56	6	20
	%		62 (48–75)	100 (96–100)	100 (63–100)	100 (79–100)	61 (42–78)	100 (94–100)	43 (18–71)	100 (83–100)
	P		<0.001		>0.99		<0.001		<0.001	
*Reported using* *Relative Numbers*	n		9	39	7	15	2	13	0	11
	%		17 (8–30)	42 (32–53)	88 (47–100)	94 (70–100)	6 (1–21)	23 (13–36)	0 (0–23)	55 (32–77)
	P		0.002		>0.99		0.08		<0.001	
*Reported using* *Absolute Numbers*	n		0	25	0	1	0	18	1	6
	%		0 (0–7)	27 (18–37)	0 (0–37)	6 (0–30)	0 (0–11)	32 (20–46)	7 (0–34)	30 (12–54)
	P		<0.001		>0.99		<0.001		0.20	

Data are number of industry and non-industry media responses that reported the indicated study characteristic, or % (95% CI).

Industry media responses were less likely than non-industry media responses to include quotes from study investigators (0/53, 0% vs 84/92, 91%; P<0.001) or other commentators (4/53, 8% vs 59/92, 64%; P<0.001), and more likely to include quotes from industry employees (53/53, 100% vs 10/92, 11%; P<0.001).

### Propagation of industry press releases

On the websites of six organizations that service, inform and advise manufacturers, marketers and retailers of dietary supplements, we identified 148 news stories that directly referenced an industry press release (Table S3). The median (range) number of news stories that referenced an industry press release per source publication was 3 (0–10). Industry press releases generated in response to 42/46 (91%) of the source publications were referenced in at least one news story on at least one of the websites.

## Discussion

Research on press releases issued by pharmaceutical companies and academic institutions has emphasized their propensity to hype research findings, by reporting positive preliminary data, omitting important study information, failing to discuss caveats and limitations, and exaggerating the importance of the results [13], [64]. We are unaware of research evaluating press releases from the supplements industry, or examining denigratory ‘spin’. Our analysis suggests that press releases issued by organizations which represent and promote the commercial interests of the manufacturers and retailers of supplemental medicines in response to the publication of clinical research findings about supplements contain more ‘spin’ than press releases and news stories from non-industry sources. Notably, the ‘spin’ in industry press releases strongly favoured use of supplements. More hyping ‘spin’ was present in industry press releases than non-industry media documents generated in response to the small number of studies reporting beneficial effects of supplements. The majority of studies (83%) that prompted industry press releases reported no benefit or harm of the supplement. In response to these studies, industry press releases were enriched for denigratory ‘spin’, and were almost unanimously (>90%) supportive of use of the supplement.

Industry press releases were also less likely than non-industry media documents to report key study characteristics such as sample size and study outcomes. This difference was apparent only for studies which reported no effect or harm of the supplement. Failure to identify and report study outcomes and provide estimates of effect size were common techniques by which industry press releases downplayed the outcomes of studies which failed to demonstrate benefit of supplements. Industry press releases never included interviews with the authors of the source publication, and rarely included opinions on the findings of the source publication from non-industry experts.

Press releases influence news stories [10], [11] and news stories influence health behaviors [65]–[67]. We found evidence for propagation of supplements industry press releases by organizations whose primary function is to inform and advise the manufacturers, marketers and consumers of dietary supplements. It is likely that these news stories influenced the attitudes of retailers and marketers of supplements, and thereby the behaviors of supplements consumers. The news stories may also have directly influenced consumer behaviour. Most supplements users do not take the agents on the advice of a health care professional [1], and many do not discuss supplement use with their doctor [4], [5], while information sources such as people other than health professionals, the internet, magazines, and news stories are influential [2], [5]. It is therefore likely that the propagation of the ‘spin’-enriched industry press releases contributes to the ongoing, and even burgeoning, enthusiasm for use of supplements in the face of accumulating evidence of their ineffectiveness [6], [58], [68] and, in some cases, harm [6], [38]. Continued use of interventions which are promoted as having health benefits despite evidence for lack of efficacy may discourage use of interventions for which evidence for efficacy is established [14] and can have adverse financial consequences for individual users [2].

Our study has limitations. Although our analyses were of press releases from three prominent supplements industry organizations, the results might not apply to press releases from other industry organizations. Media document content analysis and interpretation were subjective, but were performed independently by two reviewers, with a high level of agreement, and consensus to resolve disagreements. A blinded analysis would be ideal but, because of substantial differences in style and formatting among the sources of media documents, it was not possible to achieve without altering the documents so much that the original structure and tone would be lost. The control set of media documents included news stories and press releases, but sensitivity analyses found similar results after exclusion of the former documents. Analyses of responses to studies reporting benefits of supplements were limited by the small sample size.

Our results suggest that press releases issued by the supplements industry in response to clinical research on its products consistently include ‘spin’ that promotes the use of supplements, regardless of the research findings. Journalists, health practitioners and advisors, and consumers of supplemental medicines should therefore be sceptical of the content of press releases issued by organizations representing the supplements industry in response to clinical studies of supplements, and seek information from non-industry sources where the use of ‘spin’ is less common.

## Supporting Information

Table S1 Design and outcome of studies that generated industry press releases.
(DOCX)

Table S2 Frequency of the most common ‘spin’ techniques observed in press releases or news stories.
(DOCX)

Table S3 Propagation of industry press releases by organizations that service manufacturers and retailers of supplements.
(DOCX)

Text S1 **Data Abstraction Form 1 - Source Publications.**
(DOCX)

Text S2 **Data Abstraction Form 2 - Press Releases and New Stories.**
(DOCX)

Text S3 **‘Spin’ assessment.**
(DOCX)
