# Supplementary material for: Press Releases Issued by Supplements Industry Organisations and Non-Industry Organisations in Response to Publication of Clinical Research Findings: A Case-Control Study
Source: PLoS One. 2014 Jul 3;9(7):e101533. doi: 10.1371/journal.pone.0101533 (PMC4081644; doi:10.1371/journal.pone.0101533)
Supplement: Table S1 — Design and outcome of studies that generated industry press releases. (DOCX) [file pone.0101533.s001.docx]

Table S1. Design and outcome of studies that generated industry press releases

| **Study Design** | **Study Outcome** | | |
| --- | --- | --- | --- |
|  | **Benefit** | **No effect** | **Harm** |
| Randomized controlled trials | 3 | 22 | 1 |
| Meta-analysis of randomized controlled trials | 2 | 4 | 5 |
| Observational studies | 3 | 2 | 4 |
| Total | 8 | 28 | 10 |
