# Supplementary material for: Press Releases Issued by Supplements Industry Organisations and Non-Industry Organisations in Response to Publication of Clinical Research Findings: A Case-Control Study
Source: PLoS One. 2014 Jul 3;9(7):e101533. doi: 10.1371/journal.pone.0101533 (PMC4081644; doi:10.1371/journal.pone.0101533)
Supplement: Table S2 — Frequency of the most common ‘spin’ techniques observed in press releases or news stories. (DOCX) [file pone.0101533.s002.docx]

Table S2. Frequency of the most common ‘spin’ techniques observed in press releases or news stories. Data are n, or median (95% CI**)**

|  | **Industry (n=53)** | | **Non-industry (n=92)** | | **P** |
| --- | --- | --- | --- | --- | --- |
|  | **n** | **% (95% CI)** | **n** | **% (95% CI)** |  |
| ***Hyping ‘Spin’ Techniques*** |  |  |  |  |  |
| Adamant or dogmatic language and conclusions | 13 | 25 (14-38) | 10 | 11 (5-19) | 0.04 |
| Side-tracking - shifting focus away from source article | 13 | 25 (14-38) | 5 | 5 (2-12) | 0.001 |
| Focus on subgroup analysis | 11 | 21 (11-34) | 9 | 10 (5-18) | 0.08 |
| Side-tracking - shifting focus from study endpoints to endpoints not evaluated | 8 | 15 (7-28) | 5 | 5 (2-12) | 0.07 |
|  |  |  |  |  |  |
| ***Denigratory ‘Spin’ Techniques*** |  |  |  |  |  |
| Adamant or dogmatic language and conclusions | 39 | 74 (60-85) | 18 | 20 (12-29) | <0.0001 |
| Dismissal of findings in favour of previously published body of evidence | 37 | 70 (56-82) | 22 | 24 (16-34) | <0.0001 |
| Side-tracking - shifting focus away from source article | 37 | 70 (56-82) | 14 | 15 (9-24) | <0.0001 |
| Erroneous statements about trial methodology or results | 28 | 53 (39-67) | 7 | 8 (3-15) | <0.0001 |
| Side-tracking – shifting focus from study endpoint(s) to endpoints not evaluated | 22 | 42 (28-56) | 11 | 12 (6-20) | <0.0001 |
| Focus on perceived methodological flaws – study population incorrect/inappropriate | 21 | 40 (26-54) | 11 | 12 (6-20) | 0.0003 |
| Failure to identify the study outcomes | 20 | 38 (25-52) | 0 | 0 (0-4) | <0.0001 |
| Focus on perceived methodological flaws – failure to provide intervention correctly | 17 | 32 (20-46) | 10 | 11 (5-19) | 0.003 |
| Denigratory/dismissive language towards investigator, editorialist or the journal of publication | 11 | 21 (11-34) | 5 | 5 (2-12) | 0.01 |
| Focus on perceived methodological flaws (meta-analysis) – study inclusion | 10 | 19 (9-32) | 10 | 11 (5-19) | 0.21 |
| Side-tracking – shifting focus from neutral/ adverse efficacy endpoint(s) to safety endpoint(s) | 9 | 17 (8-30) | 2 | 2 (0-8) | 0.002 |
| Special pleading | 8 | 15 (7-28) | 8 | 9 (4-16) | 0.28 |
