# Supplementary material for: Press Releases Issued by Supplements Industry Organisations and Non-Industry Organisations in Response to Publication of Clinical Research Findings: A Case-Control Study
Source: PLoS One. 2014 Jul 3;9(7):e101533. doi: 10.1371/journal.pone.0101533 (PMC4081644; doi:10.1371/journal.pone.0101533)
Supplement: Table S3 — Propagation of industry press releases by organizations that service manufacturers and retailers of supplements. (DOCX) [file pone.0101533.s003.docx]

Table S3. Propagation of industry press releases by organizations that service manufacturers and retailers of supplements

| **Organization** | **Function or purpose** | **News stories that reference industry press releases (n)** |
| --- | --- | --- |
| NewHope360 | “the premiere digital marketplace that connects the healthy lifestyle industry from supply to shelf”^a^ | 53 |
| Nutritional Outlook | “a leading resource for the manufacturers of dietary supplements and healthy food and beverages”^b^ | 22 |
| Nutraceuticals World | “our priority is to serve the manufacturers of dietary supplements, functional foods and nutritional beverages”^c^ | 21 |
| Natural Products Insider | “the #1 print, online and e-mail resource for marketers, manufacturers and formulators of dietary supplements”^d^ | 30 |
| Whole Foods Magazine | “..editorial focus is, and always has been, on informing and educating retailers about how to improve their businesses”^e^ | 6 |
| Drug Store News^1^ | “..the leading publication in print and online covering the business of retail pharmacy.”^f^ | 16 |

^1^ News stories only available from 4/1/2007

^a^ <http://newhope360.com/>

^b^ <http://www.nutritionaloutlook.com/>

^c^ <http://www.nutraceuticalsworld.com>

^d^ <http://www.naturalproductsinsider.com/>

^e^ <http://www.wholefoodsmagazine.com/>

^f^ <http://drugstorenews.com/>
