# Supplementary material for: Press Releases Issued by Supplements Industry Organisations and Non-Industry Organisations in Response to Publication of Clinical Research Findings: A Case-Control Study
Source: PLoS One. 2014 Jul 3;9(7):e101533. doi: 10.1371/journal.pone.0101533 (PMC4081644; doi:10.1371/journal.pone.0101533)
Supplement: Text S1 — Data Abstraction Form 1 - Source Publications. (DOCX) [file pone.0101533.s004.docx]

Text S1. Data Abstraction Form 1 - Source Publications

Reader ID: ………… Article #: ……………

1. Original Article**:**

1.1. Title: ……………………………………………………………..…............................

1.2. First author: ……………………………………………………………..….................

2. Publication journal**:**

2.1. Type:  General journal  Specialised journal

2.2. Impact factor: ………………………………………………………………………....

2.3. Country of publication: ……………………………………….....................................

2.4. Name of Journal: ☐ NEJM ☐ JAMA

☐ Lancet ☐ Ann Int Med

☐ PLOS Med ☐ BMJ

☐ Arch Int Med ☐Other: ……………...…

3. Date of print publication: (dd/mm/yyyy):

3.1 Print Publication: …………………………….

3.2 Online publication: …………………………..

4. Trial Registration number:  Reported  Not reported

5. Sample size: …………….

6. Study design:  RCT  Meta-RCTs

Observational  Meta-Observational

7. Primary endpoint(s):

…………………………………………………………………………………………...…

….………………………………………………………………………………………..…

……………………………………………………………………………………………...……………………………………………………………………………………………...

8. Secondary endpoint(s):

…………………………………………………………………………………………...…

….………………………………………………………………………………………..…

……………………………………………………………………………………………...……………………………………………………………………………………………...

9. Experimental treatment/exposure

9.1. Name: ……………………………………………………..…………………………..

9.2. Type of Prevention:  Primary Prevention  Secondary Prevention

10. Comparator:  Placebo/ control intervention

Usual care / no treatment

Active treatment

Other: ………………………………………………...

Unclear

11. Funding source:  Profit  Non-profit

Both  None

Not reported

12. Conflicts of Interest:  Reported  Not reported

13. Type of primary outcome:  Efficacy  Safety

Both  Unclear

14. Study conclusions:  Favours supplement use

Doesn’t favour supplement use – no benefit

Doesn’t favour supplement use – harm

*For Question 14, refer to the conclusions section of abstract, if uncertain, search main text.*

15. Industry Responses:  CRN  NPA

ANH

16. Non-industry Responses:  NIH: ……………………………………...……..……

Journal of Publication: ………………..…………..…

News Agency 1: …………………..………………....

News Agency 2: ………………..……………………
