# Supplementary material for: Press Releases Issued by Supplements Industry Organisations and Non-Industry Organisations in Response to Publication of Clinical Research Findings: A Case-Control Study
Source: PLoS One. 2014 Jul 3;9(7):e101533. doi: 10.1371/journal.pone.0101533 (PMC4081644; doi:10.1371/journal.pone.0101533)
Supplement: Text S2 — Data Abstraction Form 2 - Press Releases and New Stories. (DOCX) [file pone.0101533.s005.docx]

Text S2 – Data Abstraction Form 2 - Press Releases and New Stories

Reader ID: ………… Article #: ………

1. Source:  CRN  NPA  ANH

NIH: ……………………………………………………….....……..

Journal of Publication: …………………………………………...…

News Agency 1: …………………………………………………….

News Agency 2: …………………………………………….………

2. Sample Size:

2.1. Does the media document provide the sample size? ☐ Yes ☐ No
2.2. If yes, what is the sample size? …………………………..

3. Primary Outcome:

3.1. Is the primary outcome clearly identified as such? ☐ Yes ☐ No

3.2. How is the primary outcome/result described? (Mark all that apply)

☐ Primary outcome/result described in words

☐ Primary outcome/result described in relative numbers

☐ Primary outcome/result described in absolute numbers

☐ Primary outcome/result described in another numerical form

☐ No outcome/result mentioned

4. Describe the overall direction of the study finding, as assessed by reading the media document, with regard to the primary exposure/intervention of interest. (Mark all that apply)

☐ Good news (something helpful)

☐ No difference (no effect)
☐ Bad news (something harmful)
☐ No intervention (not applicable)

☐ Cannot conclude

5. What type of study was the study described as?

☐ Study design was not described.

☐ An RCT

☐ A meta-analysis of RCTs

☐ An observational study

☐ A meta-analysis of observational studies

☐ A meta-analysis, trial design unspecified

☐ Other: ……………………………………

6. If the study involved an intervention, how were side effects/harms described? (Mark all that apply)

☐ Side effects/harms described in words

☐ Side effects/harms described in relative numbers

☐ Side effects/harms described in absolute numbers

☐ Side effects/harms described in another numerical form

☐ No side effects/ harms mentioned

☐ Study does not involve an intervention

7. Does the media document use the term ‘significant’? (Mark all that apply)

☐ Clearly in terms of statistical significance

☐ Clearly in terms of clinical significance

☐ Uses the term ‘significant’ but context is ambiguous

☐ Does not use the term ‘significant’

8. If the outcome is a surrogate marker, is there a caveat mentioned?

☐ Mentioned

☐ Explicit warning

☐ Not mentioned

☐ Not a surrogate (not applicable)

9. Does the media document mention any flaws/caveats regarding study design/conduct?

***MUST STATE IN THE CONTEXT OF BEING A CAVEAT***

9.1. Confounders:  Mentioned  Explicit warning

Not mentioned  Not applicable (RCT)

9.2. Uncontrolled:  Mentioned  Explicit warning

Not mentioned  Not applicable (controlled)

9.3. Other flaws:  Mentioned  Explicit warning

Not mentioned  Not applicable

10. Are any study funding sources stated?  Yes  No

11. Are there any statements about conflicts of interest?  States COI

States no COI

No statement

12. Industry Staff**:**

12.1. Does the media document name industry staff?  Yes  No

12.2. If industry staff named, record names:

……………………………………………………………………………………………

……………………………………………………………………………………………

……………………………………………………………………………………………

12.3. Does the media document quote industry staff?  Yes  No

12.4. If industry staff quoted, extract quotes:

……………………………………………………………………………………………

……………………………………………………………………………………………

……………………………………………………………………………………………

12.5. Do any quotes hype the presented results? ☐ Yes ☐ No
12.6. Do any quotes denigrate/dismiss the presented results? ☐ Yes ☐ No

13. Investigator:

13.1. Does the media document contain quote(s) or statements attributed to the investigator(s)?  Yes  No

13.2. Do any quotes hype the presented results? ☐ Yes ☐ No
13.3. Do any quotes denigrate/dismiss the presented results? ☐ Yes ☐ No

14. Independent Experts:

14.1. Does the media document contain quote(s) or statements attributed to independent experts?  Yes  No

14.2. Do any quotes hype the presented results? ☐ Yes ☐ No
14.3. Do any quotes denigrate/dismiss the presented results? ☐ Yes ☐ No

15. Does the media document facilitate access to the full article?

Provides study title

Provides full citation

Provides electronic link to article

Does not facilitate access to source publication

16. What view does the media document offer on the veracity or believability of the study findings?

Supportive view

Non-supportive view

No view offered

17. Interpretation of Media Document Title:

| 17.1. | Does the title leave you with the impression that all or some patients should: | ☐ Definitely obtain the experimental treatment evaluated  ☐ Probably obtain the experimental treatment evaluated  ☐ Patients should decide for themselves (the title was neutral)  ☐ Probably not obtain the experimental treatment evaluated  ☐ Definitely not obtain the experimental treatment evaluated  ☐ Cannot conclude |
| --- | --- | --- |
|  |  |  |
| 17.2. | Does the title leave you with the impression that there is: | ☐ No uncertainty about the benefit (or lack of benefit) of the experimental treatment evaluated  ☐ A little uncertainty about the benefit (or lack of benefit) of the experimental treatment evaluated  ☐ A lot uncertainty about the benefit (or lack of benefit) of the experimental treatment evaluated  ☐ Cannot conclude |

18. Interpretation of Media Document Full Text:

| 18.1. | Does the full text leave you with the impression that all or some patients should: | ☐ Definitely obtain the experimental treatment evaluated  ☐ Probably obtain the experimental treatment evaluated  ☐ Patients should decide for themselves (the media document was neutral)  ☐ Probably not obtain the experimental treatment evaluated  ☐ Definitely not obtain the experimental treatment evaluated  ☐ Cannot conclude |
| --- | --- | --- |
|  |  |  |
| 18.2. | Does the full text leave you with the impression that there is: | ☐ No uncertainty about the benefit (or lack of benefit) of the experimental treatment evaluated  ☐ A little uncertainty about the benefit (or lack of benefit) of the experimental treatment evaluated  ☐ A lot uncertainty about the benefit (or lack of benefit) of the experimental treatment evaluated  ☐ Cannot conclude |

19. Overall, does the media document hype the study findings? ☐ Yes ☐ No

20. Overall, does the media document denigrate the study findings? ☐ Yes ☐ No
