# Supplementary material for: Press Releases Issued by Supplements Industry Organisations and Non-Industry Organisations in Response to Publication of Clinical Research Findings: A Case-Control Study
Source: PLoS One. 2014 Jul 3;9(7):e101533. doi: 10.1371/journal.pone.0101533 (PMC4081644; doi:10.1371/journal.pone.0101533)
Supplement: Text S3 — ‘Spin’ assessment. (DOCX) [file pone.0101533.s006.docx]

Text S3. ‘Spin’ assessment

If the response to Question 19 (Data Abstraction form 2) was Yes, analyse for **hyping ‘spin’**

| a | No acknowledgment of non-statistically significant primary outcomes | Yes | No |
| --- | --- | --- | --- |
| b | Claiming equivalence when results failed to demonstrate a statistically significant difference | Yes | No |
| c | Focus on positive/negative secondary outcome | Yes | No |
| d | Focus on subgroup analysis | Yes | No |
| e | Focus on within-group comparison | Yes | No |
| f | Non-statistically significant outcome reported as if it was significant | Yes | No |
| g | Ignored safety data | Yes | No |
| h | Inadequate claim of safety | Yes | No |
| i | Inappropriate extrapolation to other populations | Yes | No |
| j | Inappropriate extrapolation to other endpoints | Yes | No |
| k | Side-tracking - shifting focus away from source article | Yes | No |
| l | Side-tracking - shifting focus from study endpoints to endpoints not evaluated | Yes | No |
| m | Inferring causality from an observational study | Yes | No |
| n | Argument by anecdote | Yes | No |
| o | Adamant or dogmatic language and conclusions | Yes | No |
| p | Erroneous statements about trial methodology or results | Yes | No |

q Other hyping ‘spin’ techniques:

………………………………………………………………………………………………………………………………………………………………………………………………………………………………………………………………………………………………………………………………………………………………………………………………………………………………………………………………………….………………………………………………………………………………………………………………………………………………………………………………………………

r Recurrent themes/ideation:

………………………………………………………………………………………………………………………………………………………………………………………………………………………….………………………………………………………………………………………………………………………………………………………………………………………………………………………….…………………………………………………………………………………………………………………………………………………………………………………………………………………

**Hyping ‘spin’ score:** ..........

*Hyping ‘spin’ score is given by the number of yes responses from question a - p and the number of responses given to question q. If the response to question 16 (Data abstraction form 2) is No, then the hyping ‘spin’ score is 0.*

If the response to question 20 (Data Abstraction form 2) was Yes, analyse for **denigratory ‘spin’**

| a | Dismissal of findings in favour of previously published body of evidence | Yes | No |
| --- | --- | --- | --- |
| b | Incorrect interpretation of levels of evidence e.g. promotion of observational study findings over those of RCTs | Yes | No |
| c | Argument by anecdote | Yes | No |
| d | Special pleading, eg intervention cannot be assessed by standard research methodologies, intervention must be administered as part of a ‘package’ of treatment | Yes | No |
| e | Focus on perceived methodological flaws – sample size | Yes | No |
| f | Focus on perceived methodological flaws – study population considered to be incorrect/inappropriate | Yes | No |
| g | Focus on perceived methodological flaws (meta-analysis) – study inclusion | Yes | No |
| h | Focus on perceived methodological flaws – important baseline variable(s) not measured | Yes | No |
| i | Focus on perceived methodological flaws – failure to control for dietary variables during trial | Yes | No |
| j | Focus on perceived methodological flaws – failure to provide intervention correctly (dose/type/combination with other intervention) | Yes | No |
| k | Adamant or dogmatic language and conclusions | Yes | No |
| l | Erroneous statements about trial methodology or results | Yes | No |
| m | Side-tracking – shifting focus away from source article | Yes | No |
| n | Side-tracking – shifting focus from study endpoint(s) to endpoints not evaluated | Yes | No |
| o | Side-tracking – shifting focus from neutral/ adverse efficacy endpoint(s) to safety endpoint(s) | Yes | No |
| p | Denigratory/ dismissive language towards investigator, editorialist and the journal of publication. | Yes | No |
| q | Failure to identify the study outcomes. | Yes | No |

r Other dismissive/ denigratory ‘spin’ techniques:

…………………………………………………………………………………………………………………………………………………………………………………………………………………………

…………………………………………………………………………………………………………………………………………………………………………………………………………………………

……………………………………………………………………………………………………………

s Recurrent themes/ideation:

………………………………………………………………………………………………………………………………………………………………………………………………………………………

…………………………………………………………………………………………………………………………………………………………………………………………………………………………

**Denigratory ‘spin’ score:** ..........

*Denigratory ‘spin’ score is the number of yes responses from question a – q and the number of responses given to question r. If the response to question 17 (Data abstraction form 2) is No, then the denigratory ‘spin’ score is 0.*
